# The relation between amyotrophic lateral sclerosis and inorganic selenium in drinking water: a population-based case-control study

**DOI:** 10.1186/1476-069X-9-77

**Published:** 2010-12-06

**Authors:** Marco Vinceti, Francesca Bonvicini, Kenneth J Rothman, Luciano Vescovi, Feiyue Wang

**Affiliations:** 1 CREAGEN - Environmental, Genetic and Nutritional Epidemiology Research Center, University of Modena and Reggio Emilia, Reggio Emilia, Italy; 2 Department of Public Health, Local Health Unit of Reggio Emilia, Reggio Emilia, Italy; 3 RTI Health Solutions, Research Triangle Park, NC, USA; 4 Department of Epidemiology, Boston University School of Public Health, Boston, MA, USA; 5 Laboratory of Environmental Chemistry, IREN, Reggio Emilia, Italy; 6 Department of Environment and Geography & Department of Chemistry, University of Manitoba, Winnipeg, Canada

## Abstract

**Background:**

A community in northern Italy was previously reported to have an excess incidence of amyotrophic lateral sclerosis among residents exposed to high levels of inorganic selenium in their drinking water.

**Methods:**

To assess the extent to which such association persisted in the decade following its initial observation, we conducted a population-based case-control study encompassing forty-one newly-diagnosed cases of amyotrophic lateral sclerosis and eighty-two age- and sex-matched controls. We measured long-term intake of inorganic selenium along with other potentially neurotoxic trace elements.

**Results:**

We found that consumption of drinking water containing ≥ 1 μg/l of inorganic selenium was associated with a relative risk for amyotrophic lateral sclerosis of 5.4 (95% confidence interval 1.1-26) after adjustment for confounding factors. Greater amounts of cumulative inorganic selenium intake were associated with progressively increasing effects, with a relative risk of 2.1 (95% confidence interval 0.5-9.1) for intermediate levels of cumulative intake and 6.4 (95% confidence interval 1.3-31) for high intake.

**Conclusion:**

Based on these results, coupled with other epidemiologic data and with findings from animal studies that show specific toxicity of the trace element on motor neurons, we hypothesize that dietary intake of inorganic selenium through drinking water increases the risk for amyotrophic lateral sclerosis.

## Background

Amyotrophic lateral sclerosis (ALS), a severe neurodegenerative disease, has no established environmental risk factors [1]. Although some studies report that its incidence has been stable, others report changes in incidence over time and geographic variation in occurrence [2,3]. Some environmental factors, particularly neurotoxic elements and pesticides, have been implicated by some investigations [4-6]. Two epidemiologic studies [7,8] have focused on the possible role of selenium (Se), an element of nutritional and toxicological interest [9]. Environmental Se exists in organic and inorganic forms, with the organic forms being virtually the only ones found in food [10]. Dietary Se intake in Italy has been estimated to be about 50 μg/d per person [11]. Inorganic forms of Se are found more commonly in drinking water or some occupational settings. A safe intake range for Se is currently under debate [9]. Recent observations imply that it might be much lower than the value that has been assumed [12,13].

In an earlier study, Vinceti et al. reported that the population of Reggio Emilia, northern Italy, where some inhabitants consumed drinking water with unusually high Se content, experienced excess ALS incidence during the period 1986-1994, with a relative risk of 4.2 compared with those consuming water with lower levels of Se [8]. The present study was undertaken to determine whether this association has persisted during the years since the earlier report.

## Methods

We conducted a population-based case-control study in the Reggio Emilia municipality, measuring Se levels in the drinking water of cases and matched controls and using interviews to collect other relevant data from study subjects or their proxies. Eligible cases were all Reggio Emilia residents who received a first-time diagnosis of ALS during the years 1995 to 2006, provided that they had been residents of Reggio Emilia for at least six months. Cases were identified using a methodological approach that has already been described in detail [3]. In collaboration with expert neurologists, we reviewed the Hospital Discharges Register of the Emilia Romagna Region for both inpatients and outpatients of public and private hospitals from 1995 to 2006, as well as death certificates from 1996 through 2007, and all prescriptions for riluzole, the only drug specific for ALS. Only patients fulfilling the El Escorial revised diagnostic criteria [14] for probable or definite ALS, and residing in the Reggio Emilia municipality at the time of diagnosis, were included as cases.

We selected controls from the general population of Reggio Emilia, identifiable through annual directories of residents made available by the General Registry Office of the region. Using the calendar-year specific file of municipal residents corresponding to the year of diagnosis for each case, we randomly selected two controls matched to the case for year of birth and sex, using the *sample *command of Stata statistical software (version 10.1, Stata Corp., College Station, TX 2009).

We contacted all cases and controls by phone or visiting them directly, and we asked permission to administer a questionnaire at the subject's home, at the University office in Reggio Emilia, or in a few instances by telephone. All cases or their relatives were contacted jointly by two of the coauthors (M.V. and F.B.) and all but one agreed to participate. For deceased cases (39/41) and controls (13/82), we asked permission to administer the questionnaire to the closest available relative, in most cases the marital partner or a son or daughter. All participants were able to provide detailed information about the subject's drinking water consumption and provided well water samples if applicable. We were unable to enroll eleven of the initially sampled eighty-two controls: one could not be contacted, six refused to participate, and four could not provide samples of well-water owing to the collapse of the well coupled with absence of other wells in the vicinity. For these eleven subjects, we resampled the population to obtain substitute controls.

The questionnaire we administered was designed to collect information about residential history and sources of domestic drinking water during the thirty-five years before diagnosis for cases, and for the corresponding period for the matched controls. The questionnaire also asked about consumption of dietary supplements (types and duration), family history of ALS in first-degree relatives, occupational history, life-style factors (smoking habits, coffee and alcohol consumption), and history of trauma sufficient to result in admission to a hospital. We also checked residential history information reported by the subject against the files of the Municipal Registry Office. When the latter data were inconsistent with those recalled by the subjects during the interview, we contacted the subject to resolve the discrepancy.

For study participants who reported consuming well water, we obtained details, including year starting, year ending, and estimated percentage of total water consumed. We also sought permission to sample this water, when it was available. If subjects were no longer residing in the house that had the well, we contacted those currently living at that address and we asked their permission to sample the water. For three study subjects (all controls), the original well was not accessible in 2009 because it had collapsed, but after contacting a neighboring family we were able to get a sample of water from a nearby well. We ascertained that the well depth from the neighboring well was comparable (± 5 m) with that of the subject's original well and that the distance between the two wells was <20 m.

Water samples were acidified with a 65% nitric acid solution (2 mL/100 mL sample) and analyzed for concentrations of potentially neurotoxic elements (aluminium, arsenic, chromium, cadmium, copper, iron, lithium, manganese, lead, selenium and zinc) by inductively coupled plasma mass spectrometry (ICP-MS), using an Agilent 7500ce spectrometer equipped with an Octopole Reaction System with a Micromist nebulizer. For all water samples with Se ≥1 μg/l, we carried out Se speciation analysis following a procedure reported in detail elsewhere [15]. The Se speciation was determined on a Waters 626 high performance liquid chromatograph (HPLC) (Waters, USA) interfaced with an Elan DRC-II ICP-MS (PerkinElmer, Canada). Samples were injected manually through a Rheodyne Model 9125 injector equipped with a 100 μL PEEK sample loop with a non-metallic pump. The analytical column was an IonPac^® ^AS18 anion-exchange column (250 mm × 4 mm i.d.; Dionex, USA), with an IonPac^® ^AG18 as a guard column. The mobile phase was a 23 mM KOH. Injection volume was fixed at 100 μL and the mobile phase was delivered at 1 mL/min isocratically. ^82^Se was monitored on the ICP-MS under the standard mode and the chromatographic peaks were quantified by area with the software Chromera 1.2 (PerkinElmer, Canada).

The concentration of trace elements in the public water supply system in the Reggio Emilia municipality has been uniform throughout the system and steady during the study period, as verified by historical data on water composition provided by the local Municipal Water Supply Agency (named IREN). The only exception to this constancy has been Se itself, which was unusually high (about 8 μg/l [16]) in the tap water distributed to a small district, "Rivalta", in the period 1972-88. Before 1972 and after 1988, the water supplied to Rivalta was the same as that supplied to the rest of Reggio Emilia, but during this period water supplied to Rivalta residents came from two local wells having only one distinctive chemical characteristic, a high Se content. We sampled this water again for Se speciation for this study. We assigned a Se concentration of 8 μg/l to municipal tap water consumed by subjects residing in Rivalta for at least six months during 1972-88 [16,17]. We assigned a value of 0 μg of Se for all other consumption of municipal water, as the concentration of Se in the tap water never otherwise reached the detection limit of the analytical methodology.

We then computed an estimate of overall Se intake through drinking water during the 35 years before the diagnosis date (or corresponding date for controls). We derived this total by multiplying the number of days of exposure within the 35 year period by 2.6 liters of water each day (the estimate yielded by a survey among pregnant women in an area very close to the study one [18]), and by the Se concentration in the water that was being consumed on the day we measured it.

We also ascertained information on occupational exposure to pesticide, industrial chemicals and electromagnetic fields by reviewing the occupational history of study subjects and estimating the occurrence of such exposure for at least six months. We estimated the relative risk (RR) of ALS following Se exposure through drinking water from Mantel-Haenszel odds ratios in a stratified analysis, and from odds ratios estimated from conditional logistic regression models that included the potential confounders.

## Results

During the study period, 42 new cases of ALS occurred in the Reggio Emilia municipal population. One patient refused to participate. The distribution of study subjects by age, sex, educational level, occupational history and case or control status is given in Table 1. No subject reported any consumption of Se-containing supplements.

**Table 1:** Characteristics of cases and controls, population-based case-control study on ALS, Reggio Emilia, northern Italy, 1995-2006

Characteristics	Cases n (%)	Controls n (%)
**Sex**		
Male	30 (73)	60 (73)
Female	11 (27)	22 (27)
**Age**		
40-54	9 (22)	18 (22)
55-69	13 (32)	26 (32)
≥70	19 (46)	38 (46)
**Educational level**		
Elementary or lower	20 (49)	38 (46)
Middle school	7 (17)	26 (32)
High school	10 (24)	15 (18)
University	4 (10)	3 (4)
**Occupational exposure**		
Pesticides	13 (32)	11 (13)
Chemicals	13 (32)	26 (32)
Electromagnetic fields	1 (2)	0 (0)

Most study subjects had consumed water from the municipal system that was low in Se. Three cases and four controls consumed the high Se municipal tap water that was distributed from 1972-88 in the Rivalta district [16]. None of these study subjects consumed Rivalta municipal water for less than six months. Eleven cases and ten controls reported consuming at least 75% of their drinking water from private wells. We collected samples from all 21 wells.

The concentration of trace elements (Al, Cd, Cu, Fe, Li, Mn, Pb, Zn, Cr and As) in well water samples was similar for cases and controls. We compared their concentration (considering both municipal tapwater and wellwater) to the usual dietary intake from recent Italian studies [19,20], except for As, for which we used foreign data owing to unavailability of national data [21], and for inorganic Se itself, which we assumed to be virtually absent in foods [10]. For all elements apart from inorganic Se and lithium (Li), intake from drinking water was negligible compared with usual intake through diet (Table 2). Se in all the water samples was almost exclusively present as inorganic Se, in the form of hexavalent Se (selenate), the Se species found in the Rivalta municipal tapwater during 1972-88 period [16].

**Table 2:** Estimated daily intake (μg/day) of trace elements through drinking water and through diet in study population

Element	Centile of estimated intake through tapwater^a^			Estimated intake through diet
		
	5^th^	50^th^	95^th^	
Al	0.0	0.0	7.8	3580
As	0.0	0.0	1.0	62
Cr	0.3	5.5	5.5	104.5^b ^
Cd	0.0	0.0	0.0	13.6
Cu	2.9	10.7	13.5	1140
Fe	39.5	39.5	759.2	11000
Li	20.3	20.3	92.0	29.9
Mn	4.2	4.2	190.6	1380
Pb	0.2	4.2	4.2	55.2
Se^c^	0.0	0.0	20.8	0
Zn	54.1	54.1	3861.0	12000

^a^Expressed as 5th, 50th and 95th percentiles, assuming a consumption of wellwater or municipal tapwater of 2.6 l/day

^b^Average of median values in males and females

^c^Inorganic Se only

**Figure 1:**
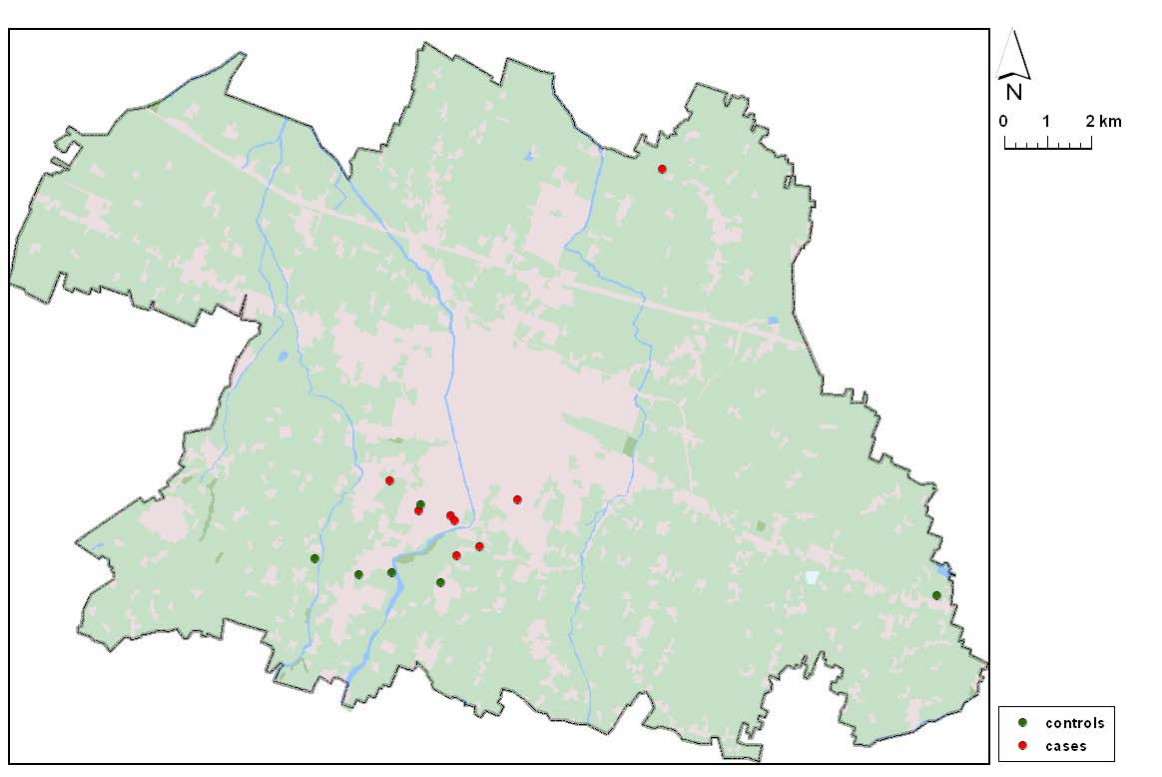
**Map of the Reggio Emilia municipality, northern Italy (extension 231.6 km^2^, population 169,223 at September 30, 2010), showing the locations where ALS cases and controls consumed high-Se drinking water in the 1995-2006 period**.

Overall, eight cases and six controls consumed high levels of Se (≥ 1 μg/l) in their drinking water (Figure 1), whatever the source, either municipal water supply system or a private well, yielding a crude RR estimate of 3.1 (95% CI 1.0 - 9.6) and an age- and sex-adjusted Mantel-Haenszel RR estimate of 3.3 (95% CI 1.0 - 11.0) (Table 3). Because these results do not take into account the matched design, these estimates are biased toward 1.0 [22]. When the matching was taken into account using a conditional logistic regression model, the RR estimate was 4.2 (95% CI 1.1 - 16, Table 4). Among males, the RR estimate was 4.3 (95% CI 0.8 - 23), and it was 4.0 (95% CI 0.4 - 44) among females. Adding educational attainment level, exposures to pesticide, industrial chemicals and electromagnetic fields to the conditional logistic model did not substantially change the results (Table 4). When we carried out an analysis using three levels of lifetime intake of inorganic Se through drinking water, 0, <10.000 and ≥10.000 μg, the RR estimates by level were 1.0 (referent category), 2.1 (95% CI 0.5-9.1) and 6.4 (95% CI 1.3-31), respectively (Table 5).

**Table 3:** Age- and sex-adjusted Mantel-Haenszel relative risk (RR-MH) with 95% confidence interval (CI) of amyotrophic lateral sclerosis according to long-term consumption of drinking water with Se content ≥1 μg/l vs. <1 μg/l, Reggio Emilia municipality, northern Italy, 1995-2006

		Males		Females		Total	
		
Age		High Se	Low Se	High Se	Low Se	High Se	Low Se
40-54	cases	1	6	1	1	2	7
	controls	1	13	0	4	1	17
							
55-69	cases	3	6	0	4	3	10
	controls	4	14	0	8	4	22
							
≥70	cases	2	12	1	4	3	16
	controls	0	28	1	9	1	37
							
Total	cases	6	24	2	9	8	33
	controls	5	55	1	21	6	76
RR-MH 3.3 (95% CI 1.0 - 11)							

**Table 4:** Relative risk (RR) with 95% confidence interval (CI) of amyotrophic lateral sclerosis according to long-term consumption of drinking water with Se content ≥1 μg/l vs. <1 μg/l in conditional logistic regression models, Reggio Emilia municipality, northern Italy, 1995-2006

Model	RR	95% CI
Bivariate model	4.2	1.1 - 16
Multivariate model - 1^a^	4.3	1.1 - 17
Multivariate model - 2^b^	5.4	1.3 - 24
Multivariate model - 3^c^	5.4	1.1 - 26

^a^Adjusting for educational attainment level

^b^Adjusting for occupational pesticide exposure

^c^Adjusting for all potential confounders (educational attainment level and exposure to pesticides, industrial chemicals and electromagnetic fields)

**Table 5:** Relative risk (RR) with 95% confidence interval (CI) of amyotrophic lateral sclerosis, according to overall lifetime estimated intakea of inorganic Se through drinking water, Reggio Emilia municipality, northern Italy, 1995-2006

**Lifetime Se intake (μg)**	**Cases/controls**	**RR**	**95% CI**
0	27/68	1.0 (referent)	**-**
1-9999	6/7	2.1	0.5-9.1
≥10000	8/7	6.4	1.3-31

^a^Estimated on the basis of drinking water consumption during the 35-year period before diagnosis in patients and the corresponding period for matched referents.

In high doses Li may induce neurological abnormalities such as dysarthria, ataxia, tremor and impaired cognitive functions [23]. When we dichotomized consumption of Li at the median drinking water Li concentration of 7.8 μg/l, we observed an elevated RR (2.7, 95% CI 0.8 - 9.5). The RR for Li, however, decreased to 2.2 (95% CI 0.6-8.3) after adjusting for Se water concentrations above 1.0 μg/l and to 1.6 (95% CI 0.4-6.5) after adjusting for cumulative lifetime inorganic Se intake. The effect of Se intake remained strongly associated with ALS risk even when controlling for Li intake (RR 4.3, 95% CI 1.1-17). Results were similar when we used a different measure of Li exposure, the estimated lifetime intake (RR 4.6, 95% CI 1.1-19). None of the other trace elements in drinking water analyzed in the study resulted to be associated with increased ALS risk.

## Discussion

We observed a strong association, with an apparent dose-response relation, between consumption of drinking water containing inorganic Se and risk of ALS in our study population. These findings reinforce previous observations made in the same locale. In this study, we included drinking water exposure to other minerals in addition to Se exposure.

Apart from an ALS cluster in South Dakota reported by Kilness and Hochberg [7], there is epidemiologic evidence from China suggesting that high levels of exposure to environmental Se deleteriously affect motor neuron function in humans [9,24]. In a village in the Enshi prefecture whose inhabitants had a high prevalence of Se toxicosis from naturally occurring Se, motor neuron system abnormalities including paralysis and hemiplegia were noted. Unfortunately, little epidemiologic detail on the amount and sources of Se exposures and occurrence or relative risk of these outcomes was reported [25]. Subsequently, Fordyce reported the occurrence of motor neuron abnormalities such as 'tingling limbs', 'no strength in limbs and 'paralysis' in 180 individuals from the same area affected by Se intoxication, on the basis of data kept by local public health officials [26].

In the present study, ALS risk was associated with consumption of drinking water with a Se content lower than the current allowable level of 10 μg/l set by the World Health Organization [27]. That standard, however, was set on the basis of the very weak epidemiologic evidence available on the overall health effects of Se [9,27,28]. No report relates any chronic disease risk to Se content in drinking water, apart from our current findings and the earlier observations from the Rivalta area of Reggio Emilia [8,16,17]. Moreover, recent laboratory observations on rat sciatic nerve fibers showed that Se neurotoxicity may occur at levels of exposure as low as 0.8 μg/l [29], and human neuron cells have been recently shown to be considerably more sensitive to the toxicity of low doses of Se compared with other cell types [30]. This level is lower than those likely experienced by exposed subjects in the current study, based on the estimated relation between Se intake and resulting blood levels, which has been suggested to be in the order of 1,5 μg/l per 1 μg of daily Se intake [31]. Other laboratory studies confirmed that toxic effects of inorganic Se (selenate and selenite) occur at extremely low concentrations [32], comparable with those attributable to our Se-exposed subjects. Finally, it has been suggested that toxic effects of Se may be cumulative, being related to overall lifetime exposure and not just to short-term exposure [33].

Pesticides have been reported to be associated with ALS risk in some, but not all studies [4,6]. Pesticide exposure did not appear to be an important confounding factor in this study, nor was smoking, coffee and alcohol consumption, history of trauma, intense physical activity and family history of ALS. A limitation of these analyses is that information on these variables was self-reported and thus subject to inaccurate recall, although such inaccuracies could not plausibly explain our primary finding.

Support for a link between Se exposure and ALS comes from some veterinary and laboratory studies. Administration of inorganic Se and to some extent organic Se induces poliomyelomalacia and polioencephalomalacia in swine, including a selective degeneration of the anterior horns of the spinal cords [34-37]. Motor neuron abnormalities have also been described in cattle grazing in seleniferous areas [28], and experimental observations appear to confirm such association [38]. Following Se treatment, a decrease in locomotor activity in rats [39] and hind limb paralysis and cardiorespiratory effects in mice [40] have been observed. In laboratory studies of inorganic and less frequently organic Se in different animal species, the trace element has shown to induce several toxic effects that might lead to motor neuron toxicity [41-44]. Se has also been shown to damage muscle function in animal models and to inhibit axonal conduction and excitatory postsynaptic potentials [9,45]. In a human neuron cell line, Se compounds were recently shown to induce a pattern of biochemical alterations which have been implicated in ALS pathogenesis [30].

Our findings are consistent with a Se-ALS relation that might be specific for the inorganic, soluble species of this element that is typically found in aquifers [15]. We caution that these results ought not be extended to the organic forms of the trace element found in foods and in Se-containing dietary supplements. Laboratory studies have consistently shown that neurotoxic effects are exclusive or greatly enhanced in the presence of inorganic Se compared with the organic forms [41,43], with a ratio of median lethal dose comparing inorganic vs. organic Se as high as 43:1 [40]. Nevertheless, experimental and observational studies in swine showed that motor neuron toxicity of Se is not entirely limited to the inorganic species of Se [34], as is the case for Se neurotoxicity in laboratory studies [30,40,41,43]. Thus, although these results should not be extended to organic Se, the possibility remains that there is some effect of organic Se exposure on the nervous system at high enough levels.

A weakness of this study is the limited size of the exposed population in this natural setting, leading to broad confidence intervals for the effect estimates. The occurrence of high Se levels in well water has recently been reported in various areas of the world [9], and these unusual environmental settings (as well as rare occupational exposures to inorganic Se compounds) may offer opportunities to investigate the Se-ALS relation. Conversely, currently available biomarkers of exposure appear to be unsuitable to study this issue, owing to their inability to reflect actual exposure to inorganic Se. Inorganic Se is retained in the body to a much lesser extent than the organic species at equivalent levels of exposure, despite its greater toxicological activity [34,46]. Another difficulty is the lack of reliable long-term indicators of Se exposure and the rapid progression of ALS.

We based our drinking water estimate of Se content on currently available Se levels for well waters and on historical data for Se municipal tap water content, thus raising the possibility of some misclassification of exposure. This misclassification would have been non-differential for cases and controls, biasing results toward the null, and thus not explaining the strong association we found. Moreover, available data for the public tap water and for the Rivalta wells (whose water Se content was measured in the 1980s) indicate that current levels are similar to the historical ones. Therefore, any exposure misclassification would have had only a small effect.

Finally, we expect that an additional source of misclassification of exposure in the study population came from the consumption of bottled water, which is currently very common in Italy [18]. Bottled water distributed in Italy has very low concentrations of Se (median value 0.1 μg/l [47]). Nevertheless, additional data collected from the fourteen subjects (or their families) reporting consumption of drinking water with Se content ≥1 μg/l identified only three cases and three controls who reported some (limited) consumption of bottled water, mostly confined to the recent past, which implies that this source of bias was limited.

## Conclusions

In conclusion, we found an excess risk of ALS associated with exposure to inorganic Se through drinking water. Given the consistency of these results with those previously obtained in the same study area and given the selective toxicity of Se on motor neurons observed in some animal studies, we hypothesize that exposure to inorganic Se through water is a cause of ALS, and encourage others to test this hypothesis with additional research.

## Abbreviations

ALS: Amyotrophic lateral sclerosis; Se: selenium; ICP-MS: inductively coupled plasma mass spectrometry; RR: relative risk; CI: confidence interval; Li: lithium.

## Competing interests

The authors declare that they have no competing interests.

## Authors' contributions

MV and FB designed the original study, and with KJR analyzed and interpreted the data, and wrote the manuscript. LV and FW carried out the analytical determinations (overall selenium content in water samples and selenium speciation). All authors read and approved the final manuscript.
